# Compositional Dependence of Phase Selection in CoCrCu_0.1_FeMoNi-Based High-Entropy Alloys

**DOI:** 10.3390/ma11081290

**Published:** 2018-07-25

**Authors:** Ning Liu, Chen Chen, Isaac Chang, Pengjie Zhou, Xiaojing Wang

**Affiliations:** 1 School of Materials Science and Engineering, Jiangsu University of Science and Technology, Zhenjiang 212003, China; cc1870806525@163.com (C.C.); zhoupengjie@just.edu.cn (P.Z.); xjwang.0@163.com (X.W.); 2 Brunel Centre for Advanced Solidification (BCAST), Brunel University, Kingston Lane, London UB8 3PH, UK; isaac.chang@brunel.ac.uk

**Keywords:** high-entropy alloy, microstructure, eutectic structure, phase selection

## Abstract

To study the effect of alloy composition on phase selection in the CoCrCu_0.1_FeMoNi high-entropy alloy (HEA), Mo was partially replaced by Co, Cr, Fe, and Ni. The microstructures and phase selection behaviors of the CoCrCu_0.1_FeMoNi HEA system were investigated. Dendritic, inter-dendritic, and eutectic microstructures were observed in the as-solidified HEAs. A simple face centered cubic (FCC) single-phase solid solution was obtained when the molar ratio of Fe, Co, and Ni was increased to 1.7 at the expense of Mo, indicating that Fe, Co, and Ni stabilized the FCC structure. The FCC structure was favored at the atomic radius ratio *δ* ≤ 2.8, valence electron concentration (VEC) ≥ 8.27, mixing entropy ΔS ≤ 13.037, local lattice distortion parameter α_2_ ≤ 0.0051, and ΔS/*δ*^2^ > 1.7. Mixed FCC + body centered cubic (BCC) structures occurred for 4.1 ≤ *δ* ≤ 4.3 and 7.71 ≤ VEC ≤ 7.86; FCC or/and BCC + intermetallic (IM) mixtures were favored at 2.8 ≤ *δ* ≤ 4.1 or *δ* > 4.3 and 7.39 < VEC ≤ 8.27. The IM phase is favored at electronegativity differences greater than 0.133. However, ΔS, α_2_, and ΔS/*δ*^2^ were inefficient in identifying the (FCC or/and BCC + IM)/(FCC + BCC) transition. Moreover, the mixing enthalpy cannot predict phase structures in this system.

## 1. Introduction

High-entropy alloys (HEAs) represent a new class of materials that have attracted extensive attention since 2004 [1,2,3,4,5,6,7,8]. HEAs are defined as alloys containing at least five major elements wherein every major element has an atomic fraction between 5% and 35% [9]. Various studies on HEAs, including composition, processing, crystal structure and microstructure, and physical and mechanical properties, have been performed in the past years [5,6,7,8,9,10,11,12,13,14,15,16,17,18,19,20,21,22,23].

The microstructures and crystalline phases present in HEAs are very sensitive to the alloy composition. An α phase appears when the molar ratio of Mo in AlCoCrFeNiMo*_x_* alloys exceeds 0.1 [17]. Changes in the molar ratio of Fe, Co, and Cr in AlCoCrFeMo_0.5_Ni alloys affect the crystalline phases and mechanical properties. As the Mo content increases, the volume fraction of the σ phase increases in Ni_2_CrFeMo*_x_* alloys [18]. However, the volume fraction of the σ phase is increased with increasing Cr content. Eventually, the dendritic matrix of AlCoCr*_x_*FeMo_0.5_Ni HEAs is changed from the ordered B2 phase to the σ phase [19]. As the Co content in AlCo*_x_*CrFeMo_0.5_Ni HEAs changes from *x* = 0.5 to *x* = 2.0, the phase changes from BCC to BCC + FCC + σ, respectively. Recently, it was found that the precipitation of intermetallic (IM) compounds of σ and μ phases could strengthen CoCrFeNiMo_0.3_ alloy without causing serious embrittlement [21]. Moreover, the ordered B2 solid-solution and σ phases were presented in FeAlCrNiMo*_x_* HEAs with increasing Mo content [22]. Most previous investigations have focused on the effects of the addition or content change of one or two elements on the microstructure and properties of the HEA. However, studies on the effect of each individual element on the microstructure and phase selection have not yet been reported. Additional systematic research will be necessary in the near future to guide the exploration of HEAs.

Many parameters are related to phase selection in HEAs, including the atomic radii differences (*δ*), differences in electronegativity (ΔX), the valence electron concentration (VEC), the enthalpy of mixing (ΔH_mix_), and the mixing entropy (ΔS_mix_) [24,25,26,27,28,29,30,31]. Based on these parameters, many criteria have been proposed for phase prediction in HEAs. Zhang et al. summarized a solid-solution phase-forming rule using *δ*, ΔH_mix_, and ΔS_mix_ with *δ* ≤ 6.6%, −22 ≤ ΔH_mix_ ≤ 7 kJ/mol, and 11 ≤ ΔS_mix_ ≤ 19.5 J/(K·mol) [24]. To limit the target of discussion to simple disordered phases, the conditions are more strict: *δ* ≤ 4.3%, −15 ≤ ΔH_mix_ ≤ 5 kJ/mol, and 12 ≤ ΔS_mix_ ≤ 17.5 J/(K·mol). Guo proposed that the phase stability of the FCC and BCC solid solution was correlated with VEC; for VEC < 6.87 the BCC phase was stable; for VEC > 8, FCC was [25]. Later, the stability of the σ phase was studied and it was predicted that alloys with 6.88 ≤ VEC ≤ 7.84 were prone to σ phase formation [30]. However, this criterion works well only for Cr- and V-containing HEAs. Recently, complex ordered phases were found to be stable for ΔX > 0.133, except for HEAs containing a large amount of Al [31]. More recently, Wang et al. [32] proposed a new parameter, α_2_, to address the local lattice distortion of crystalline lattices in HEAs. This parameter effectively explained the lattice distortion, intrinsic strain energy, and excess entropy in HEAs.

As mentioned above, the phases present in HEAs are remarkably dependent on the alloy composition. In a previous work, the CoCrCu_0.1_FeMoNi alloy exhibited the duplex microstructure of BCC + FCC [33]. Under compositional change, the microstructural behavior is uncertain: the HEA could retain this simple solid solution mixture or intermetallic (IM) phases could appear. In order to address this question, it is necessary to study the effect of alloy composition on the phase selection of CoCrCu_0.1_FeMoNi alloys. Hence, the present work investigates the partial substitution of Mo by Cr, Co, Ni, and Fe. The effect of the relative contents of Cr, Co, Ni, Fe, and Mo on the microstructure and crystal structures of CoCrCu_0.1_FeMoNi-based HEAs was studied in this work in order to understand the phase selection mechanism in this alloy system.

## 2. Materials and Methods

The proposed HEAs were prepared via vacuum arc melting in a Ti-gettered Ar atmosphere with subsequent melt solidification in a water-cooled Cu crucible. A mixture of the appropriate amounts of the constituent elements with purities > 99.9 wt % for each alloy was flipped and melted at least four times to ensure thorough chemical homogeneity. Table 1 shows the compositions prepared in this study. As-cast samples were then sectioned and polished for microstructural and compositional characterization using scanning electron microscopy (SEM, JEOL-5410, JEOL Ltd., Tokyo, Japan), energy dispersive X-ray spectrometry (EDS, JEOL Ltd., Tokyo, Japan), and an X-ray diffractometer (XRD, Rigaku ME510-FM2, Rigaku Ltd., Tokyo, Japan) at a scanning speed of 4°/min and a scanning range from 30° to 100° using a Cu target and an applied voltage and current of 30 kV and 20 mA, respectively.

## 3. Results

### 3.1. Co_a_CrCu_0.1_FeMo_2−a_Ni Alloys

**Figure 1:**
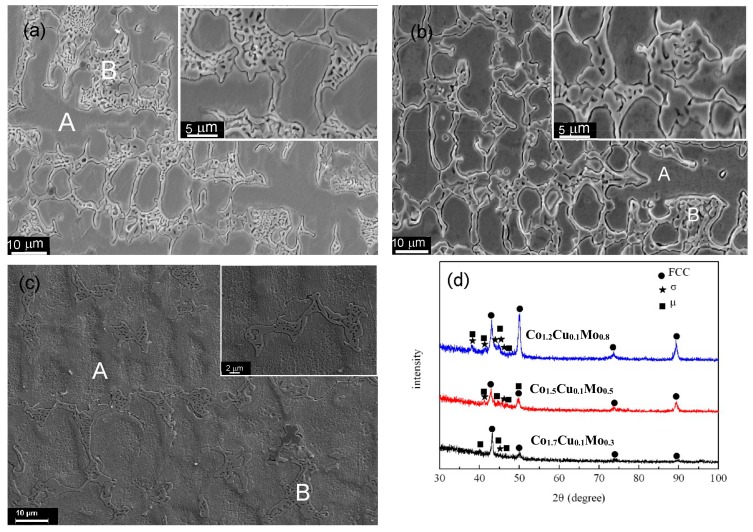
The microstructures and phases of Co*_a_*CrCu_0.1_FeMo_2−*a*_Ni HEAs. (**a**) Co_1.2_CrCu_0.1_FeMo_0.8_Ni; (**b**) Co_1.5_CrCu_0.1_FeMo_0.5_Ni; (**c**) Co_1.7_CrCu_0.1_FeMo_0.3_Ni; (**d**) X-ray diffractometer (XRD) patterns.

The microstructures of the Co*_a_*CrCu_0.1_FeMo_2−*a*_Ni (*a* = 1.2, 1.5 and 1.7) alloys are shown in Figure 1. A typical eutectic structure is found in the inter-dendritic region, and the volume fraction of the eutectic mixture is decreased with increasing Co and decreasing Mo. Table 2 shows the actual composition and contents of different regions in the microstructures, as detected by EDS. The dendrites are enriched in Co, Cu, Fe, and Ni, while the contents of Cr and Mo are higher in the inter-dendritic region B. The composition of the inter-dendritic eutectic region B is approximately (CrMo)_54_(CoCuFeNi)_46_ according to EDS. This means that the content of Cr and Mo is 54% and that of Co, Cu, Fe, and Ni is 46%. Furthermore, the Cu content in the inter-dendritic region is increased with decreasing Mo content. This is related to the positive ΔH_mix_ between Cu and Mo (+19 kJ/mol).

Figure 1d shows the XRD patterns of the Co*_a_*CrCu_0.1_FeMo_2−*a*_Ni HEAs. The FCC, σ, and μ phases are detected. The crystal structure of the σ phase is tetragonal with the lattice constants of *a* = 0.885 nm and *c* = 0.459 nm, and the σ phase is similar to the binary Co_2_Mo_3_ phase. The μ phase is tetragonal with lattice constants of *a* = 0.7381 nm and *c* = 1.8504 nm, and probably Co_7_Mo_3_ or Fe_7_Mo_6_. Both σ and μ are topologically close-packed (TCP) phases. Obviously, the volume fractions of the σ and μ phases, represented by peaks in the range of 40–50°, are decreased as the Co content increases and Mo decreases. According to the EDS and XRD results, we can identify the dendrites as the FCC phase, while σ and μ are eutectic structures.

### 3.2. CoCr_b_Cu_0.1_FeMo_2−b_Ni Alloys

**Figure 2:**
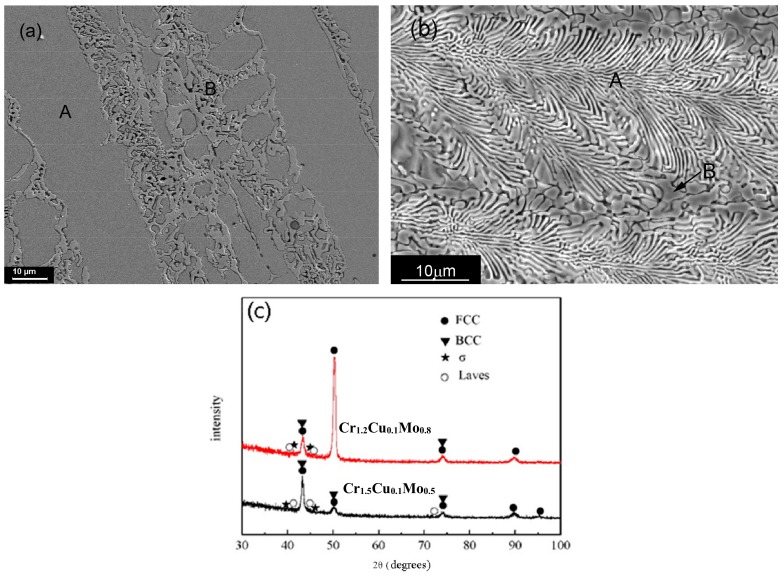
The SEM micrographs and phase structures of CoCr*_b_*Cu_0.1_FeMo_2−*b*_Ni alloys. (**a**) CoCr_1.2_Cu_0.1_FeMo_0.8_Ni; (**b**) CoCr_1.5_Cu_0.1_FeMo_0.5_Ni; (**c**) XRD patterns.

The microstructures of CoCr*_b_*Cu_0.1_FeMo_2−*b*_Ni (*b* = 1.2 and 1.5) alloys are shown in Figure 2. Dendrites and inter-dendritic regions remain in the CoCr_1.2_Cu_0.1_FeMo_0.8_Ni alloy (referred to as Cr_1.2_Cu_0.1_Mo_0.8_). The dendrites are enriched in Fe and Ni, and the Cr content in the dendrites is increased (A in Figure 2a), while the inter-dendritic region (B) is enriched with Co and Mo. However, for the CoCr_1.5_Cu_0.1_FeMo_0.5_Ni alloy (referred to as Cr_1.5_Cu_0.1_Mo_0.5_), a fully eutectic structure is found, indicating that the alloy has a eutectic composition, probably of (CrMo)_52_(CoCuFeNi)_48_, according to region A in Table 2 as detected by EDS. This means that the content of Cr and Mo is 52%, and that of Co, Cu, Fe, and Ni is 48%.

It is apparent that CoCr*_b_*Cu_0.1_FeMo_2−*b*_Ni (*b* = 1.2 and 1.5) alloys contain FCC, BCC, and TCP phases according to the XRD patterns, as shown in Figure 2c. The TCP phases correspond to the tetragonal σ phase (*a* = 0.917 nm, *c* = 0.474 nm) and a hexagonal close-packed (HCP) Laves phase (*a* = 0.473 nm, *c* = 0.772 nm). As can be seen in Figure 2c, the volume fraction of the BCC phase increases with increasing Cr, which enhances the formation of BCC phase in CoCr*_b_*Cu_0.1_FeMo_2−*b*_Ni alloys (*b* = 1.2 and 1.5). With increasing Cr and decreasing Mo, the BCC phase appears, and the volume fraction of both TCP phases decreases. According to the EDS and XRD results, the dendrites should be FCC, while the eutectic structures include BCC, σ, and Laves phases.

### 3.3. CoCrCu_0.1_Fe_c_Mo_2−c_Ni Alloys

**Figure 3:**
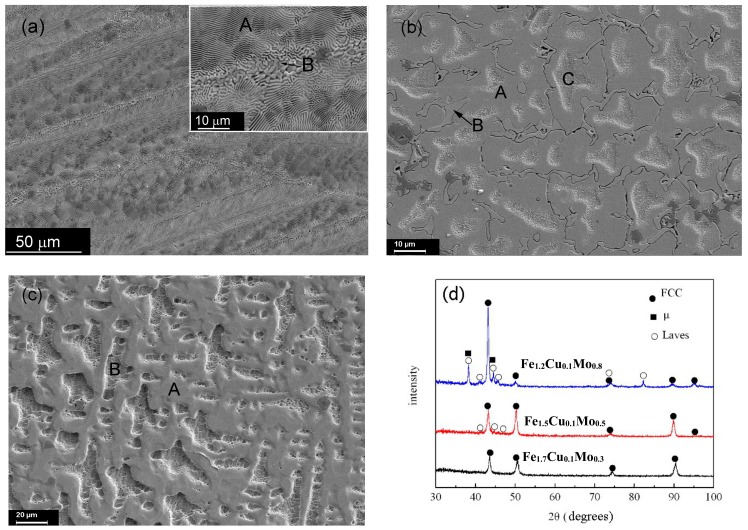
Micrographs and XRD patterns of CoCrCu_0.1_Fe*_c_*Mo_2−*c*_Ni HEAs: (**a**) Cu_0.1_Fe_1.2_Mo_0.8_; (**b**) Cu0.1Fe_1.5_Mo_0.5_; (**c**) Cu_0.1_Fe_1.7_Mo_0.3_; (**d**) XRD patterns.

Figure 3 presents the microstructures of the CoCrCu_0.1_Fe*_c_*Mo_2−*c*_Ni (*c* = 1.2, 1.5 and 1.7) HEAs. A fully eutectic structure is obtained in the CoCrCu_0.1_Fe_1.2_Mo_0.8_Ni alloy (referred to as Cu_0.1_Fe_1.2_Mo_0.8_). As shown in Table 2, the composition of the eutectic region is approximately (CrMo)_51_(CoCuFeNi)_49_. Similar to the Co_1.5_Cu_0.1_Mo_0.5_ and Co_1.7_Cu_0.1_Mo_0.3_ alloys, the microstructures of the CoCrCu_0.1_Fe_1.5_Mo_0.5_Ni (referred to as Cu_0.1_Fe_1.5_Mo_0.5_) and CoCrCu_0.1_Fe_1.7_Mo_0.3_Ni (referred to as Cu_0.1_Fe_1.7_Mo_0.3_) alloys comprise dendritic and inter-dendritic regions. The volume fraction of the inter-dendritic eutectic region decreases dramatically with increasing Fe and decreasing Mo contents. The dendrites (region A) of the Cu_0.1_Fe_1.5_Mo_0.5_ alloy is enriched in Co, Cu, Fe, and Ni; the content of Cr and Mo is higher in the inter-dendritic regions B and C. Region A of the Cu_0.1_Fe_1.7_Mo_0.3_ alloy is enriched in Mo; the content of Cu, Co, and Fe is higher in region B; and the contents of Cr and Ni are almost the same.

The XRD patterns of the CoCrCu_0.1_Fe*_c_*Mo_2−*c*_Ni (*c* = 1.2, 1.5, 1.7) HEAs are shown in Figure 3d. FCC, μ (Fe_7_Mo_3_), and Laves phases are found in these alloys. The μ phase is trigonal (*a* = 0.7381 nm, *c* = 18.504 nm) and the Laves phase is HCP with *a* = 0.473 nm and *c* = 0.772 nm). Based on the intensities of the diffraction peaks, decreased Mo and increased Fe contents yield decreases in the volume fractions of the μ and Laves phases and increases in that of the FCC phase. For the Cu_0.1_Fe_1.2_Mo_0.8_ alloy, the FCC, μ, and Laves phases form a eutectic structure. For the Cu_0.1_Fe_1.5_Mo_0.5_ alloy, region A is FCC, region B should be the μ (Fe_7_Mo_3_) phase, and region C is probably FCC. For the Cu_0.1_Fe_1.7_Mo_0.3_ alloy, both region A and B are FCC structures with different contents.

### 3.4. CoCrCu_0.1_FeMo_2−d_Ni_d_ Alloys

**Figure 4:**
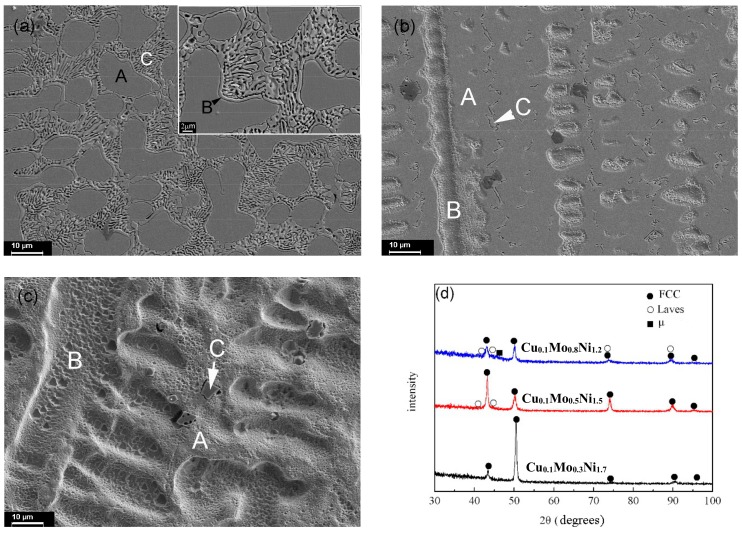
Microstructures and XRD patterns of the CoCrCu_0.1_FeMo_2*−d*_Ni*_d_* HEAs. (**a**) Cu_0.1_Mo_0.8_Ni_1.2_; (**b**) Cu_0.1_Mo_0.5_Ni_1.5_; (**c**) Cu_0.1_Mo_0.3_Ni_1.7_; (**d**) XRD patterns.

A similar microstructure, consisting of a dendritic matrix and inter-dendritic regions, is found in CoCrCu_0.1_FeMo_2−*d*_Ni*_d_* (*d* = 1.2, 1.5 and 1.7) HEAs, as shown in Figure 4. For the Cu_0.1_Mo_0.8_Ni_1.2_ alloy, the dendrites are enriched in Co, Cu, Fe, and Ni (Region A in Figure 4a), while the inter-dendritic region (B) is enriched with Cr and Mo. The composition of the eutectic region is found to be approximately (CrMo)_54_(CoCuFeNi)_46_, as shown in Table 2. For the Cu_0.1_Mo_0.5_Ni_1.5_ alloy, region A is enriched in Cu and Mo, region B has a higher content of Cr, Co, Fe, and Ni, and region C is enriched with Cr and Mo. Many flower-like structures with four petals (labeled C) are observed in the Cu_0.1_Mo_0.3_Ni_1.7_ alloy; these structures are enriched with Cr.

The XRD results demonstrate that the alloys contain a trigonal μ phase (*a* = 0.7381 nm, *c* = 18.504 nm), FCC phase, and a small amount of an HCP Laves phase (*a* = 0.473 nm, *c* = 0.772 nm), as can be seen in Figure 4d. When the molar ratio of Ni is increased to 1.7, only the FCC phase is found in the solidified microstructure. Thus, it is demonstrated that Ni promotes the formation of the FCC phase. For the Cu_0.1_Mo_0.8_Ni_1.2_ alloy, FCC is the dendritic phase, while the μ and Laves phases form a eutectic structure. For the Cu_0.1_Mo_0.5_Ni_1.5_ alloy, both regions A and B are FCC structures, and region C should be the μ phase. For the Cu_0.1_Mo_0.3_Ni_1.7_ alloy, both regions A and B are FCC structures with different contents, while region C is an unknown Cr-rich phase that cannot be detected because of its small amount.

## 4. Discussion

Two eutectic phases are found in the Cr_1.5_Cu_0.1_Mo_0.5_ and Cu_0.1_Fe_1.2_Mo_0.8_ HEAs, with probable eutectic compositions of (CrMo)_51–54_(CoCuFeNi)_46–49_. Similarly, fully eutectic structures have been obtained in CoFeNi*_x_*VMo*_y_* HEAs at both CoFeNi_1.4_VMo and CoFeNiVMo_0.6_ [14]. Recently, Lu et al. [34] have proposed a strategy to design eutectic high-entropy alloys (EHEAs) based on ΔH_mix_. They selected Zr, Nb, Hf, and Ta to replace Al in a previous AlCoCrFeNi_2.1_ EHEA, based on the relationship of ΔH_mix_ for various atomic pairs. Unfortunately, no regularities have yet been found in the current HEA system. Further research is ongoing to clarify this relationship in the future.

**Figure 5:**
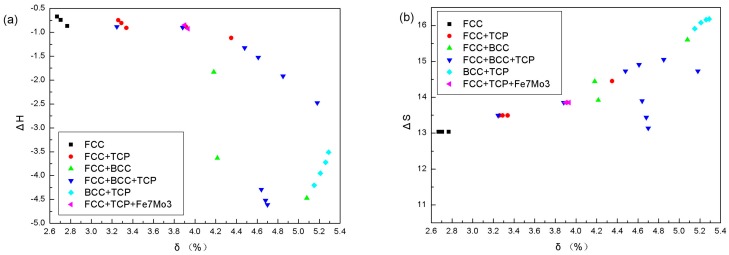

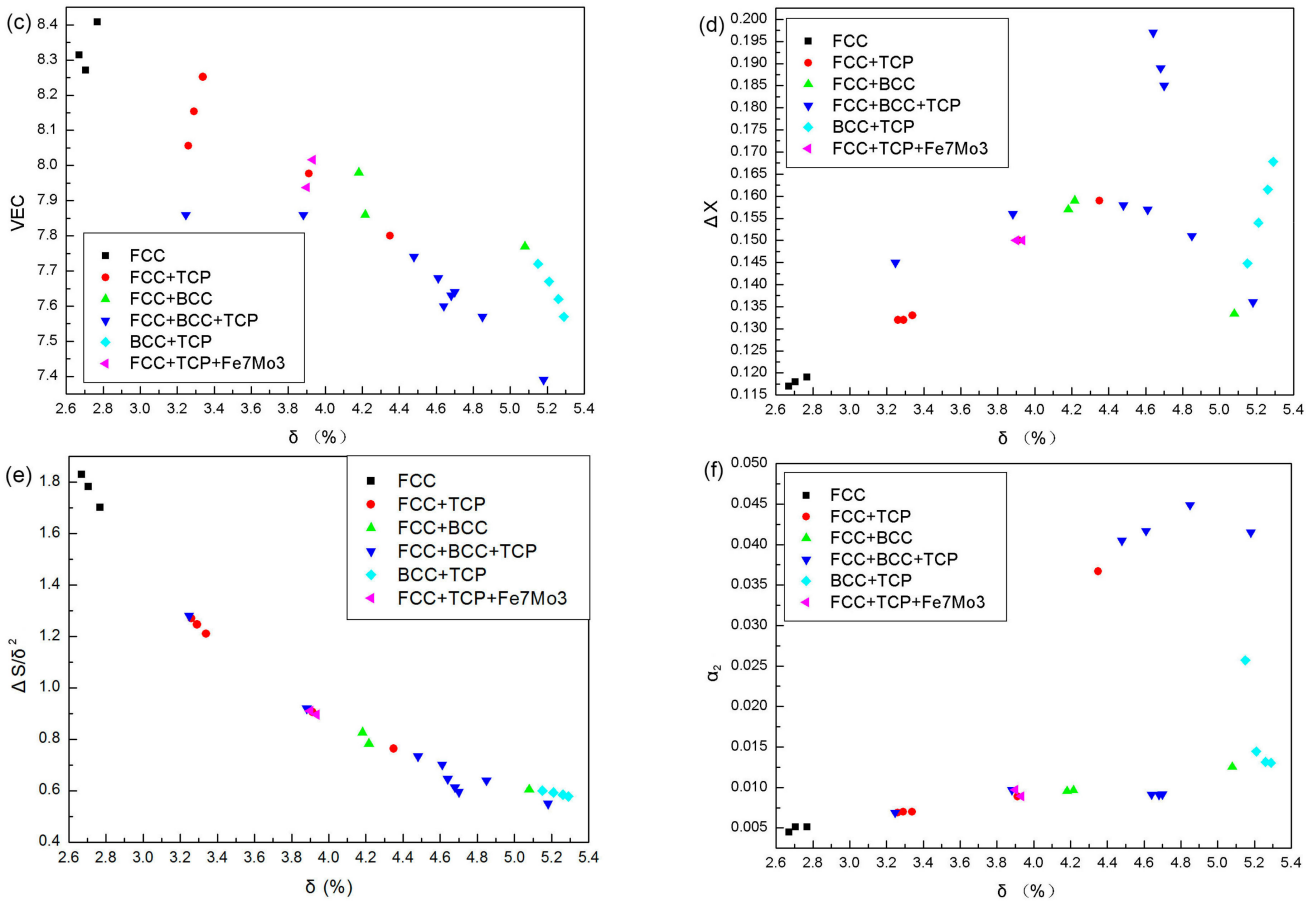
The relationships between parameters and phase structures.

The phase selection mechanism in the CoCrCu_0.1_FeMoNi-based HEAs can be understood using the parameters listed in Table 3. Based on alloy composition, a simple FCC structure is obtained only when the CoCrCu_0.1_FeMoNi-based HEAs contain higher contents of principal elements, such as Fe/Co/Ni. This suggests that Fe, Co, and Ni are FCC stabilizers in the CoCrCu_0.1_FeMoNi-based alloys. It can be found that a simple FCC structure is favorable for alloys with the smallest *δ*, ΔX, and ΔS. Conversely, alloys with large VEC values favor the formation of simple FCC structures, while TCP phases develop in alloys with smaller VEC values. TCP phases are found when the ΔH of the alloys is largely negative, with the exception of Cu_0.1_Mo_0.3_Ni_1.7_. Furthermore, alloys with small α_2_ favor the formation of a single-phase FCC structure. In the current work, the FCC structure is stable when *δ* ≤ 2.8, FCC+BCC is favored when 4.1 ≤ *δ* ≤ 4.3, and FCC or/and BCC + IM is found when 2.8 ≤ *δ* ≤ 4.1 or *δ* > 4.3, with the only exception of AlCoCrCuFeNiMo_0.2_. As shown in Figure 5c, the FCC structure is stable when VEC ≥ 8.27, but there is an overlap between the mixture types of FCC+BCC and FCC or/and BCC + IM. The IM phase is favored when ΔX > 0.133 only with the exceptions of the CoCrCu_0.1_FeMoNi and CoCrCu_0.3_FeMoNi alloys.

The results are well fitted with the criterion proposed by Lu et al. As shown in Figure 5b,f, the FCC structure is stable when ΔS ≤ 13.037 and α_2_ ≤ 0.0051; however, the (FCC+BCC)-type phase-forming ΔS and α_2_ ranges show overlaps with those of the (FCC or/and BCC + IM)-type. All the calculated values of ΔH are in the range −15 ≤ ΔH_mix_ ≤ 5 kJ/mol (Figure 5a), and except for FCC and BCC, IM phases are still found, indicating that the phase structures of the listed alloys cannot be distinguished by ΔH.

Singh demonstrated that a simple solid solution as obtained when ΔS_mix_/*δ*^2^ > 0.96, IM compounds when ΔS_mix_/*δ*^2^ < 0.24, and a mixture thereof when 0.24 < ΔS_mix_/*δ*^2^ < 0.96 [36]. As can be seen in Figure 5e, a large ΔS/*δ*^2^ value favors the formation of a single FCC phase. As the value of ΔS/*δ*^2^ decreases, more phases appear, and smaller ΔS/*δ*^2^ values favor the BCC phase. For CoCrCu_0.1_FeMoNi-based alloys, the simple FCC phase structure is favored when *Δ*S/*δ*^2^ > 1.7, while multiphase structures containing (FCC or/and BCC + IM) are found when 0.549 ≤ *Δ*S/*δ*^2^ ≤ 1.28, and the (FCC+BCC)-type phase-forming ΔS/*δ*^2^ range shows an overlap with that of the (FCC or/and BCC + IM)-type. The former famous criterion for phase-forming in HEAs cannot be used effectively in this system. Thus, new rules or parameters must be considered in the future.

## 5. Conclusions

Eutectic or dendritic microstructures were observed in as-solidified CoCrCu_0.1_FeMoNi-based HEAs. A fully eutectic microstructure was found in CoCr_1.5_Cu_0.1_FeMo_0.5_Ni and CoCrCu_0.1_Fe_1.2_Mo_0.8_Ni alloys. TCP phases were detected in most of the CoCrCu_0.1_FeMoNi-based HEAs except for the Co_1.7_CrCu_0.1_FeMo_0.3_Ni, CoCrCu_0.1_Fe_1.7_Mo_0.3_Ni, and CoCrCu_0.1_FeMo_0.3_Ni_1.7_ alloys. A simple FCC single-phase solid solution was obtained when the molar ratio of Fe, Co, and Ni was increased to 1.7 at the expense of Mo. This indicated that Fe, Co, and Ni are FCC stabilizers in the CoCrCu_0.1_FeMoNi-based alloy system.

Moreover, a simple FCC structure was found in the alloys with the smallest *δ*, ΔX, and ΔS values. Conversely, alloys with higher VEC were simple FCC structures, while TCP phases appeared to develop in alloys with decreased VEC. TCP phases were found with large negative ΔH values, with the exception of the Cu_0.1_Mo_0.3_Ni_0.7_ alloy. Furthermore, the value of α_2_ is smaller when a simple FCC structure is obtained.

For CoCrCu_0.1_FeMoNi-based alloys, the FCC structure was stable when *δ* ≤ 2.8, VEC ≥ 8.27, ΔS ≤ 13.037, α_2_ ≤ 0.0051, and ΔS/*δ*^2^ > 1.7; the mixture of FCC+BCC is favored when 4.1 ≤ *δ* ≤ 4.3 while the (FCC or/and BCC + IM) mixture is found when 2.8 ≤ *δ* ≤ 4.1 or *δ* > 4.3. IM phases are favored when ΔX > 0.133. However, some overlap remained in parameters including VEC, ΔS, α_2_, and ΔS/*δ*^2^. This indicated that these parameters are not sufficient to distinguish (FCC or/and BCC + IM) from (FCC+BCC) phase formation behaviors, and new rules or parameters must be considered for the described system. Moreover, ΔH could not predict phase structures in the current work. In summary, the phase selection behaviors in CoCrCu_0.1_FeMoNi-based HEAs can be well delineated by *δ* and ΔX.

## Figures and Tables

**Table 1:** A list of chemical composition of CoCrCu_0.1_FeMoNi-based alloys (at %).

Alloys	Co	Cr	Cu	Fe	Mo	Ni
Co_1.2_CrCu_0.1_FeMo_0.8_Ni	23.53	19.61	1.96	19.61	15.69	19.61
Co_1.5_CrCu_0.1_FeMo_0.5_Ni	29.41	19.61	1.96	19.61	9.8	19.61
Co_1.7_CrCu_0.1_FeMo_0.3_Ni	33.33	19.61	1.96	19.61	5.88	19.61
CoCr_1.2_Cu_0.1_FeMo_0.8_Ni	19.61	23.53	1.96	19.61	15.69	19.61
CoCr_1.5_Cu_0.1_FeMo_0.5_Ni	19.61	29.41	1.96	19.61	9.8	19.61
CoCrCu_0.1_Fe_1.2_Mo_0.8_Ni	19.61	19.61	1.96	23.53	15.69	19.61
CoCrCu_0.1_Fe_1.5_Mo_0.5_Ni	19.61	19.61	1.96	29.41	9.8	19.61
CoCrCu_0.1_Fe_1.7_Mo_0.3_Ni	19.61	19.61	1.96	33.33	5.88	19.61
CoCrCu_0.1_Fe Mo_0.8_Ni_1.2_	19.61	19.61	1.96	19.61	15.69	23.53
CoCrCu_0.1_Fe Mo_0.5_Ni_1.5_	19.61	19.61	1.96	19.61	9.8	29.41
CoCrCu_0.1_Fe Mo_0.3_Ni_1.7_	19.61	19.61	1.96	19.61	5.88	33.33

**Table 2:** The components of different regions in microstructures of CoCrCu_0.1_FeMoNi-based high-entropy alloys (HEAs).

Alloy	Value	Region	Co	Cr	Cu	Fe	Mo	Ni
Co*_a_*CrCu0.1FeMo_2−*a*_Ni	*a* = 1.2	Content	23.55	18.37	1.84	19.73	17.93	18.58
		A	24.17	18.46	1.85	20.45	14.32	20.75
		B	19.32	18.01	0.43	14.57	36.10	11.57
	*a* = 1.5	Content	27.92	20.60	1.8	19.63	9.5	19.55
		A	20.45	27.40	1.68	21.25	8.93	20.29
		B	17.03	33.13	0.82	16.70	19.91	12.41
	*a* = 1.7	Content	33.63	19.57	1.75	19.8	6.00	19.25
		A	34.86	17.83	1.61	20.30	6.09	19.31
		B	30.34	20.33	1.90	19.79	8.98	18.66
CoCr*_b_*Cu0.1FeMo_2−*b*_Ni	*b* = 1.2	Content	19.32	25.69	1.85	20.44	12.36	20.34
		A	18.68	30.11	-	20.20	9.53	21.47
		B	22.51	22.99	1.77	17.02	17.53	18.17
	*b* = 1.5	Content	20.02	29.45	1.51	19.23	10.36	19.43
		A	15.50	26.95	-	18.31	24.98	14.25
		B	16.76	24.25	-	19.83	19.13	20.03
CoCrCu0.1Fe*_c_*Mo_2−*c*_Ni	*c* = 1.2	Content	15.33	21.76	1.83	23.53	17.92	19.63
		A	15.70	21.66	-	20.57	29.21	12.86
		B	14.95	22.12	0.40	20.93	29.95	11.65
	*c* = 1.5	Content	17.71	21.65	1.94	29.41	10.82	18.47
		A	17.63	19.24	2.25	31.42	10.49	18.95
		B	15.51	21.98	0.34	23.16	27.19	11.83
		C	15.94	22.96	0.62	22.66	24.96	12.85
	*c* = 1.7	Content	20.8	19.98	1.64	33.30	6.05	18.23
		A	18.95	19.89	1.58	32.19	8.45	18.94
		B	22.54	20.36	1.89	34.23	2.81	18.16
CoCrCu0.1FeMo_2−*d*_Ni*_d_*	*d* = 1.2	Content	19.37	19.63	1.36	19.47	16.64	23.53
		A	19.49	18.93	1.20	21.82	13.50	25.06
		B	16.80	22.33	0.67	16.58	27.68	15.95
		C	17.03	21.79	0.45	13.97	32.56	14.20
	*d* = 1.5	Content	19.79	19.68	1.96	19.16	10.37	29.04
		A	19.09	19.13	2.15	17.77	13.22	28.65
		B	21.82	20.07	1.71	21.02	3.28	32.10
		C	18.28	22.86	0.67	15.67	21.82	20.70
	*d* = 1.7	Content	20.82	19.67	1.53	19.74	6.25	31.89
		A	17.80	21.90	1.84	18.78	8.06	31.62
		B	21.38	19.25	1.65	19.93	4.60	33.18
		C	2.44	88.74	-	3.85	0.68	4.29

**Table 3:** Phases and parameters of CoCrCu_0.1_FeMoNi-based HEAs.

Alloy	*δ* (%)	VEC	ΔX	ΔH (kJ·mol^−1^)	ΔS (J·K^−1^·mol^−1^)	α_2_	ΔS/*δ*^2^	Phases	Ref.
Co_1.2_CrCu_0.1_FeMo_0.8_Ni	3.912	7.977	0.150	−0.878	13.853	0.0089	0.9052	FCC + σ + μ	
Co_1.5_CrCu_0.1_FeMo_0.5_Ni	3.291	8.154	0.132	−0.807	13.492	0.0070	1.2457	FCC + σ + μ	
Co_1.7_CrCu_0.1_FeMo_0.3_Ni	2.705	8.271	0.118	−0.740	13.037	0.0051	1.7817	FCC	
CoCr_1.2_Cu_0.1_FeMo_0.8_Ni	3.882	7.860	0.156	−0.897	13.853	0.0097	0.9192	FCC + BCC + Laves	
CoCr_1.5_Cu_0.1_FeMo_0.5_Ni	3.247	7.860	0.145	−0.882	13.492	0.0069	1.2797	FCC + BCC + σ + Laves	
CoCrCu_0.1_Fe_1.2_Mo_0.8_Ni	3.900	7.938	0.150	−0.847	13.853	0.0097	0.9108	FCC + Laves + Fe_7_Mo_3_	
CoCrCu_0.1_Fe_1.5_Mo_0.5_Ni	3.260	8.056	0.132	−0.745	13.492	0.0069	1.2695	FCC + Laves	
CoCrCu_0.1_Fe_1.7_Mo_0.3_Ni	2.669	8.314	0.117	−0.670	13.037	0.0045	1.8301	FCC	
CoCrCu_0.1_FeMo_0.8_Ni_1.2_	3.933	8.016	0.150	−0.923	13.853	0.0089	0.8956	FCC + Laves + Fe_7_Mo_3_	
CoCrCu_0.1_FeMo_0.5_Ni_1.5_	3.339	8.252	0.133	−0.907	13.492	0.0070	1.2102	FCC + Laves	
CoCrCu_0.1_FeMo_0.3_Ni_1.7_	2.768	8.408	0.119	−0.869	13.037	0.0051	1.7016	FCC	
Al_0.1_CoCrCu_0.1_FeMo_0.9_Ni	4.35	7.80	0.159	−1.116	14.45	0.0367	0.7636	FCC + Laves	[35]
Al_0.2_CoCrCu_0.1_FeMo_0.8_Ni	4.48	7.74	0.158	−1.322	14.73	0.0405	0.7339	FCC + BCC + Laves + σ	[35]
Al_0.3_CoCrCu_0.1_FeMo_0.7_Ni	4.61	7.68	0.157	−1.523	14.91	0.0417	0.7016	FCC + BCC + Laves + σ	[35]
Al_0.5_CoCrCu_0.1_FeMo_0.5_Ni	4.85	7.57	0.151	−1.915	15.05	0.0449	0.6398	FCC + BCC+ Laves + σ	[35]
Al_0.8_CoCrCu_0.1_FeMo_0.2_Ni	5.18	7.39	0.136	−2.474	14.73	0.0415	0.5490	FCC + BCC + σ	[35]
AlCoCrCuFeNiMo_0.2_	5.08	7.77	0.133	−4.47	15.6	0.0125	0.6045	FCC + BCC	[16]
AlCoCrCuFeNiMo_0.4_	5.15	7.72	0.145	−4.2	15.91	0.0257	0.5999	BCC + α	[16]
AlCoCrCuFeNiMo_0.6_	5.21	7.67	0.154	−3.95	16.08	0.0144	0.5924	BCC + α	[16]
AlCoCrCuFeNiMo_0.8_	5.26	7.62	0.162	−3.72	16.16	0.0131	0.5841	BCC + α	[16]
AlCoCrCuFeNiMo	5.29	7.57	0.168	−3.51	16.18	0.0130	0.5782	BCC +α	[16]
CoCrCu_0.1_Fe_0.15_NiMo_1.5_Mn_0.05_	4.70	7.64	0.185	−4.61	13.14	0.0092	0.5948	FCC + BCC + μ	[13]
CoCrCu_0.1_Fe_0.15_NiMo_1.5_Mn_0.12_	4.68	7.63	0.189	−4.52	13.44	0.0091	0.6136	FCC + BCC + μ	[13]
CoCrCu_0.1_Fe_0.15_NiMo_1.5_Mn_0.3_	4.64	7.60	0.197	−4.29	13.90	0.0091	0.6456	FCC + BCC + μ	[13]
CoCrCu_0.1_FeNiMo	4.216	7.86	0.159	−3.63	13.92	0.0097	0.7831	FCC + BCC	[33]
CoCrCu_0.3_FeNiMo	4.181	7.98	0.157	−1.83	14.44	0.0096	0.8260	FCC + BCC	[33]
